# A lightweight YOLOv3 algorithm used for safety helmet detection

**DOI:** 10.1038/s41598-022-15272-w

**Published:** 2022-06-29

**Authors:** Lixia Deng, Hongquan Li, Haiying Liu, Jason Gu

**Affiliations:** 1 School of Information and Automation Engineering, Qilu University of Technology (Shandong Academy of Sciences), Jinan, 250353 Shandong Province China; 2 Department of Electrical and Computer Engineering, Dalhousie University, Halifax, NS Canada

**Keywords:** Computer science, Information technology

## Abstract

YOLOv3 is a popular and effective object detection algorithm. However, YOLOv3 has a complex network, and floating point operations (FLOPs) and parameter sizes are large. Based on this, the paper designs a new YOLOv3 network and proposes a lightweight object detection algorithm. First, two excellent networks, the Cross Stage Partial Network (CSPNet) and GhostNet, are integrated to design a more efficient residual network, CSP-Ghost-Resnet. Second, combining CSPNet and Darknet53, this paper designs a new backbone network, the ML-Darknet, to realize the gradient diversion of the backbone network. Finally, we design a lightweight multiscale feature extraction network, the PAN-CSP-Network. The newly designed network is named mini and lightweight YOLOv3 (ML-YOLOv3). Based on the helmet dataset, the FLPSs and parameter sizes of ML-YOLOv3 are only 29.7% and 29.4% of those of YOLOv3. Compared with YOLO5, ML-YOLOv3 also exhibits obvious advantages in calculation cost and detection effect.

## Introduction

Object detection is a hot research field of computer vision and digital image processing. It has been widely applied in many fields, such as unmanned driving ^1^, vehicle detection ^2^, pedestrian detection ^3^, and face recognition ^4^. Object detection is popularly used for image processing technology and tracking objects in real time. Meanwhile, object detection shows a wide range of application values in many vision tasks.

Because of the emergence of image processors with powerful computing power and large-scale data samples, deep learning has developed rapidly. Ishak Pacal et al.^5^ proposed the YOLOv3 algorithm for robust real-time polyp detection, which effectively improves the detection effect. Yizhou Chen et al.^6^ systematically explained the application of generative adversarial networks in medical image augmentation. Qiu Guan et al.^7^ applied generative adversarial networks to medical image detection to solve the problem of insufficient data samples. Kyle M ^8^ et al. adopted YOLOv3 as a detection algorithm, designing a sawtooth animal behavior analysis method. Helong Yu et al.^9^ proposed a Chinese rice variety information named entity recognition method based on a bidirectional long short-term memory network and conditional random field. This method effectively improves the identification of rice varieties. In addition, Helong Yu et al.^10^ proposed a deep learning optimization algorithm, which has significantly improved the detection of tomato pests. Deep learning used in object detection offers better generalization and robustness. The deep learning model used for self-learning object features can effectively improve the real-time performance and accuracy of object detection.

The gradual maturity of convolutional neural networks promotes the development of object detection. In 2014, Ross Girshick proposed a two-stage object detection algorithm, Region-CNN (R-CNN) ^11^, based on candidate regions. Compared with traditional object detection algorithms, R-CNN exhibits great improvement in detection effect, but there exist some problems. R-CNN produces approximately 2000 candidate regions, but there are still many redundant candidate regions. To solve problems in R-CNN, Kaiming He et al. proposed spatial pyramid pooling convolutional networks (SPPNet) in 2015 ^12^. Compared with R-CNN, SPPNet uses less convolution and reduces the reasoning time for the model. In 2015, Girshick et al. proposed Fast R-CNN ^13^ and proposed the region of interest (ROI) pooling layer based on SPPNet. Fast R-CNN is superior to SPPNet in terms of the detection effect. Under the same conditions, the model reasoning time of Fast R-CNN is approximately 8 times faster than that of R-CNN. Based on the prior experience of R-CNN and Fast RCNN, Ren et al. proposed Faster R-CNN in 2016 ^14^. It improves the comprehensive performance of the network, especially with respect to the detection speed. Meanwhile, it realizes end-to-end object detection. Combining Faster R-CNN with a fully convolutional network (FCN) ^15^, He et al. proposed Mask R-CNN in 2017 ^16^. The feature extraction part of this model adopts a feature pyramid network (FPN) ^17^ and uses an ROI alignment pooling layer instead of an ROI pooling layer. In addition, Mask R-CNN also adds the mask prediction branch. The two-stage object detection algorithm based on the candidate region achieves excellent object detection accuracy. However, due to its complex network and realization of object detection in stages, it has low detection speed and encounters difficulty in detecting objects in real time.

To improve the problems existing in the two-stage object detection algorithm, Redmon et al. proposed the one-stage object detection algorithm YOLO in 2016 ^18^. The model transforms object detection into a regression problem. YOLO no longer produces candidate regions and directly produces the location and category of the object. YOLO has a faster detection speed and realizes real-time detection. However, the detection accuracy is poor, especially for small objects. In 2017, Redmon et al. proposed YOLOv2 based on YOLO ^19^. YOLOv2 enriches the backbone feature extraction network and improves the feature extraction capacity of the network. Meanwhile, batch normalization is introduced in YOLOv2, and it effectively solves the problem of difficult convergence of nonlinear models. In addition, the anchor idea is introduced to YOLOv2 to improve the recall rate. At the same time, it is helpful to detect small objects by connecting shallow features with deep features. In 2018, Redmon et al. proposed YOLOv3 ^20^, which further enriches Darknet53. YOLOv3 adopts FPN and outputs feature maps with three different scales. The detection of objects of different sizes is realized, and the detection effect for small objects is further improved. In 2020, Bochkovskiy et al. proposed YOLOv4 ^21^ with CSPDarknet53 as the backbone network. Based on YOLOv3, some advanced excellent networks were added to YOLOv4. Spatial pyramid pooling is added at the end of the backbone network, and the path aggregation network (PANet) ^22^ replaces the multiscale feature extraction network of YOLOv3. In addition, the mosaic data enhancement method is used in data preprocessing. YOLOv4 is an efficient and powerful object detection network that is superior to YOLOv3. Relevant scholars put forward YOLOv5. Although YOLOv4 and YOLOv5 are later versions of YOLOv3, they do not abandon the original network of YOLOv3 and still adopt the overall network combining the backbone network with the multiscale feature extraction network. YOLOv3 still has high research value.

In addition to the YOLO series, Liu et al. proposed the single shot multibox detector (SSD) in 2016 ^23^. This model first proposes a multiscale feature extraction network for object detection. The shallow-level feature map has a smaller receptive field and is used to detect small objects. The deeper feature map has a large receptive field and is used to detect large objects. However, the semantic information used in shallow feature images is limited, and the detection effect of small objects is poor. Compared with two-stage object detection algorithms, one-stage object detection algorithms have faster detection speed but slightly lower detection accuracy. In recent years, object detection algorithms based on convolutional neural networks have enabled great achievements, but they are not effective for small object detection. Small objects occupy fewer pixels in the image and have lower resolution and less feature information. Meanwhile, they are easily disturbed by background noise. This makes it difficult for the feature extraction network to extract the key information of small objects. Additionally, most of the networks enlarge the receptive field through downsampling or pooling, and the size of the output feature map keeps decreasing, which may lead to feature information loss for small objects. Small object detection remains difficult.

Due to complex networks, large numbers of parameters, large physical memory and long training times, deep learning network models are difficult to apply in some mobile terminals, such as smartphones, drones or other cheap devices. In particular, the ultrahigh delay caused by limited hardware equipment has a great impact on the detection speed. The traditional convolution process increases the computational cost and wastes some computational resources. Related scholars simplify the network to achieve a lightweight network. The lightweight versions of one-stage object detection, YOLO-Tiny, YOLO-Lite, and tiny SSD, reduce the detection accuracy. Some lightweight network models have appeared in recent years, such as SqueezeNet ^24^, MobileNet ^25–27^, and ShuffleNet ^28,29^. Compared with the lightweight version of one-stage object detection, the detection effect is better. However, the detection effect is relatively low compared to the two-stage target detection algorithm. The model size of the two-stage target detection algorithm is large, and the detection speed is low. Detection accuracy and speed are two indicators that are difficult to balance. Therefore, designing a high-precision lightweight model is a hot research direction today. In addition, a lightweight network model can increase the detection effect and speed up the inferring speed of object detection in mobile terminal or cheap devices.

To solve the problem that YOLOv3 is expensive to calculate and difficult to deploy on mobile devices, this paper proposes a lightweight object detection algorithm, ML-YOLOv3, which can greatly reduce the computational cost while ensuring stable detection effort. The floating point operations (FLOPs) and parameter sizes of ML-YOLOv3 proposed in this paper are only 29.7% and 29.4% those of YOLOv3. Compared with YOLOv5, ML-YOLOv3 also has many advantages. The computational cost of ML-YOLOv3 is lower than that of YOLOv5m, but the detection effect is higher than that of YOLOv5l. Based on the helmet dataset, ML-YOLOv3 is a high-precision lightweight model with both rapid detection speed and strong detection effect. Compared to YOLOv3, the improvements we propose are more convenient for deployment on mobile devices.

The main contributions of the paper are described as follows.

1. Although the residual network can effectively extract features, a large number of traditional convolutions cause a certain degree of computing waste, which is the main reason for network complexity. Therefore, the paper integrates two excellent networks, the Cross Stage Partial Network (CSPNet) ^30^ and GhostNet ^31^, and designs a more efficient residual network, CSP-Ghost-Resnet.
2. CSPNet can effectively reduce the computational cost. We fuse the backbone network and CSPNet to design a wider backbone network. Downsampling is no longer directly connected to the residual network. Instead, CSPNet is used to split the gradient flow. Making only half the number of channels connect into the residual network further reduces the computational cost.
3. As the network deepens, the number of feature map channels increases. This also means that the multiscale feature extraction network occupies substantial computational cost. The multiscale feature extraction of YOLOv3 uses a large number of traditional convolutions, which also leads to the higher complexity of YOLOv3. This paper fuses CSP-Ghost-ResNet and PANet ^22^ and redesigns the multiscale network of YOLOv3.

## YOLOv3 methods

As a one-stage object detection algorithm, YOLOv3 transforms the detection task into a regression problem. It offers excellent detection speed and detection accuracy and is widely used in industry. YOLOv3 adopts the backbone network and multiscale feature extraction network. Darknet53 has better feature extraction abilities than Darknet19 and is better than lightweight networks such as MobileNet. The multiscale feature extraction network outputs feature maps of three different scales, which is suitable for object detection with different sizes and particularly improves the detection ability for small objects. In addition, the multiscale feature extraction network uses the idea of FPN and integrates the feature information of different sizes to effectively improve the detection effect. The Darknet53 backbone network contains a large number of residual networks, which can effectively solve the problem of gradient disappearance as the network deepens. The residual network employed by Darknet53 can be represented by Eqs. (1)–(3).

1 $$X_{1} = \sigma \left\{ {\beta \left( {W_{1} , X} \right)} \right\}$$

2 $$X_{2} = \sigma \left\{ {\beta \left( {W_{2} , X_{1} } \right)} \right\}$$

3 $$X_{3} = X + X_{2}$$

where $X$ represents an input feature, $\left( {W_{1} ,X} \right)$ represents an input feature undergoing a convolution with a weight of $W_{1}$, and the size of the convolution kernel of $W_{1}$ is 1 $\times$ 1. $\beta$ represents batch normalization, and $\sigma$ represents nonlinear ReLU activation. $\left( {W_{2} ,X_{1} } \right)$ represents an input feature undergoing a convolution with a weight of $W_{2}$, the size of the convolution kernel of $W_{2}$ is 3 $\times$ 3, $X_{2}$ represents a backbone output feature of the residual structure, and $X_{3}$ represents a final output feature of the residual network.

**Figure 1:**
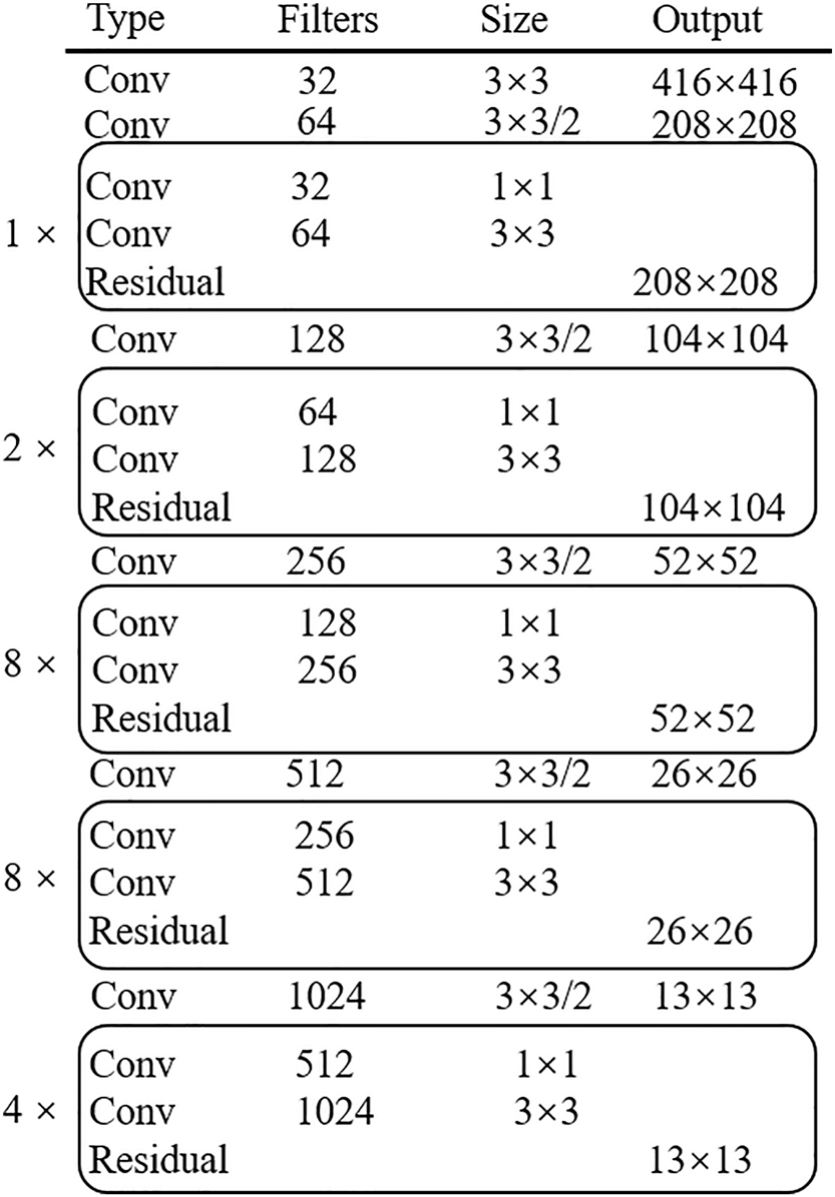
The network of Darknet53.

Darknet53 first performs a traditional 3 $\times$ 3 convolution on the input features and then stacks five residual blocks. The residual network number of each residual block is 1, 2, 8, 8, and 4. Residual blocks are connected through the convolution of downsampling. Figure 1 shows the network of Darknet53.

**Figure 2:**
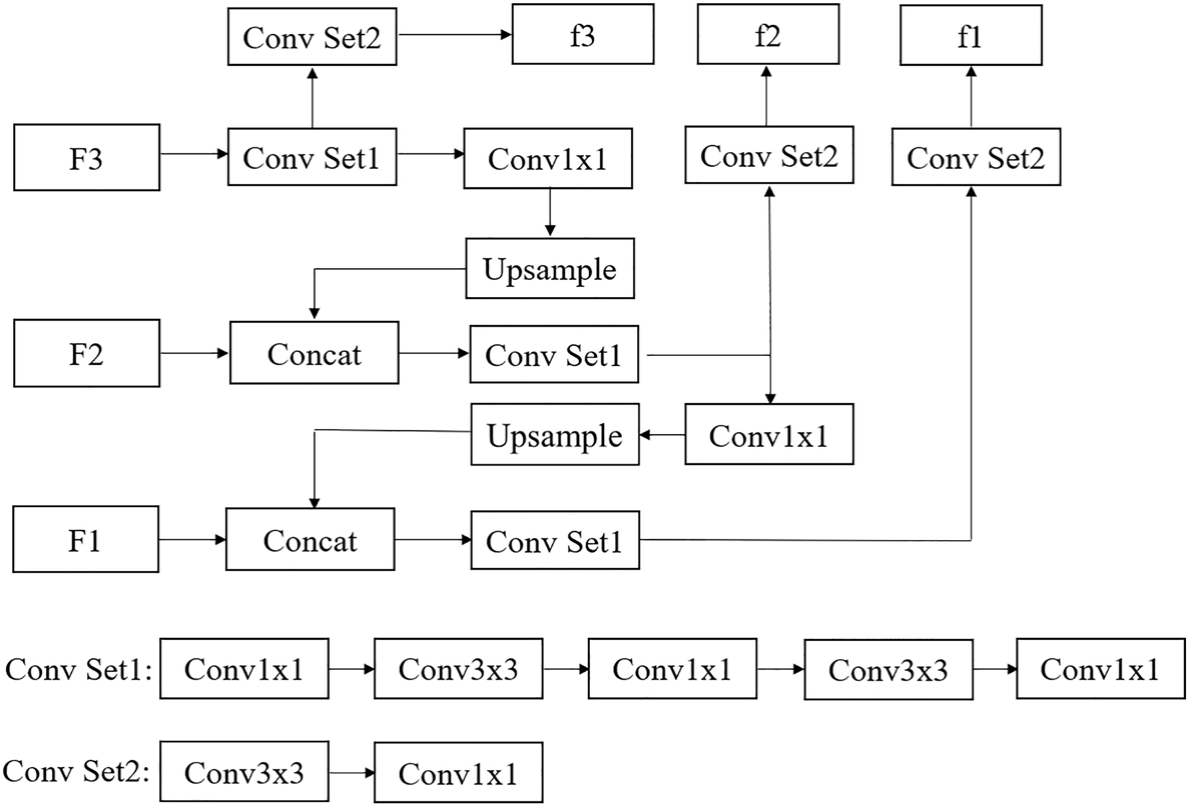
The network of multiscale feature extraction.

**Figure 3:**
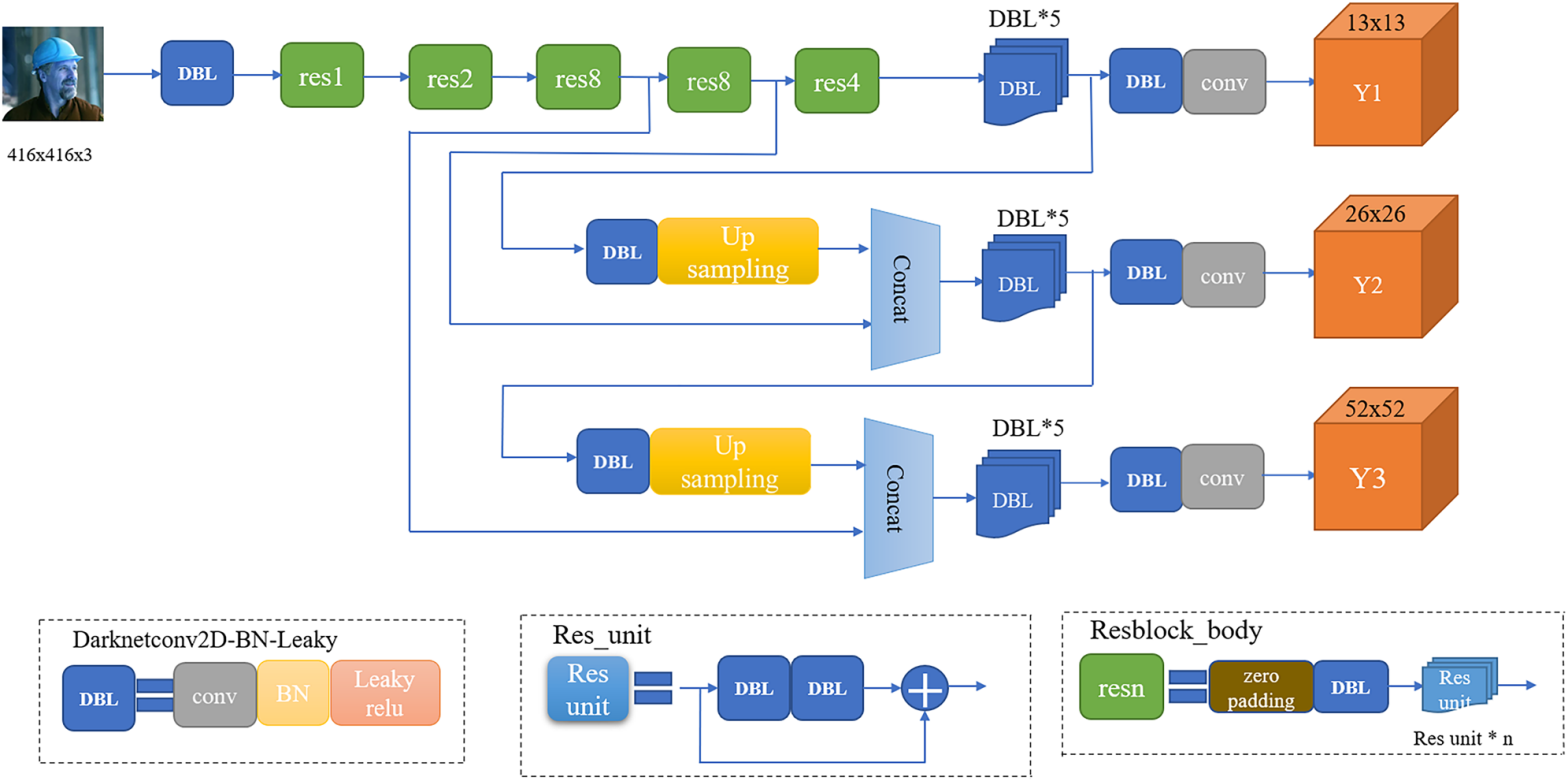
The network of YOLOv3.

FPN is mainly used to construct the network of multiscale feature extraction. The outputs of the residual blocks (the 3rd, 4th and 5th) are taken as the input of the multiscale feature extraction network. The sizes of the convolution used in the multiscale feature extraction network including upsampling are 1 $\times$ 1 and 3 $\times$ 3. Finally, the outputs include feature maps whose scales are 13 $\times$ 13, 26 $\times$ 26 and 52 $\times$ 52. Figure 2 shows the network of multiscale feature extraction. Figure 3 shows the overall network of YOLOv3.

However, YOLOv3 also poses the following disadvantages.

1. A large network leads to many model parameter sizes and more physical memory. YOLOv3 needs to rely on high-performance hardware equipment to exert its excellent performance. However, it is difficult to realize real-time detection in mobile devices or cheap devices.
2. The size of the input images of YOLOv3 is fixed. If normalizing the size of images, it is easy to cause image distortion and affect the detection effort.
3. Compared with the two-stage object detection algorithm of the RCNN series, YOLOv3 has a poorer ability to recognize the positions of objects and has a low recall rate.

## ML-YOLOv3

**Figure 4:**
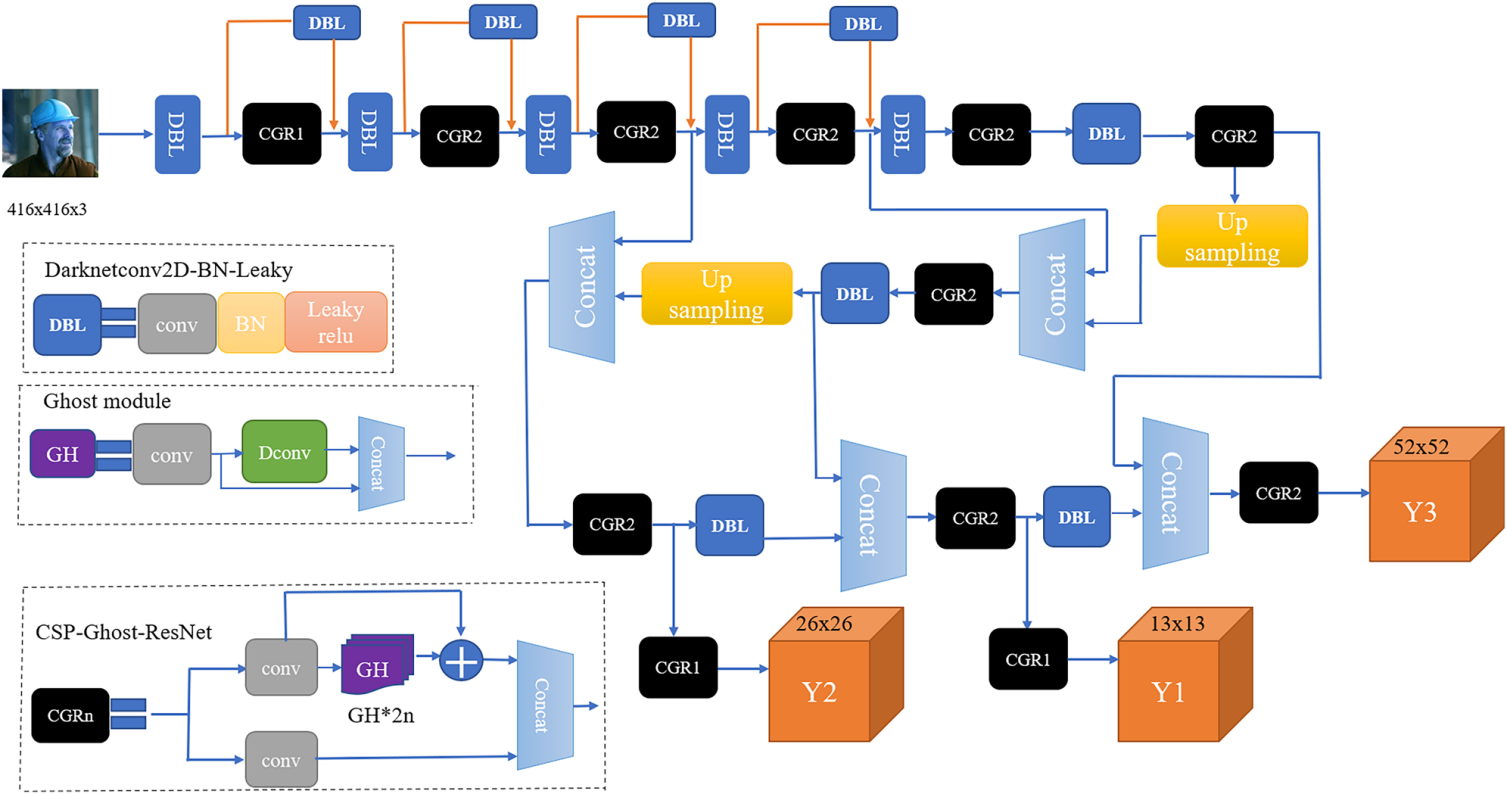
The ML-YOLOv3 network.

The paper proposes a lightweight YOLOv3 object detection algorithm. The model complexity of the proposed algorithm is greatly reduced. The paper first improves the residual module in YOLOv3 and designs a lightweight residual module by integrating the CSPNet and Ghost modules. To further reduce the computational cost, we use CSPNet to divert the gradient of the backbone network. At the same time, we redesigned a lightweight and efficient multiscale feature extraction network. It solves the problem of the complex deep network of YOLOv3. The comparison result of the ablation experiment shows that ML-YOLOv3 achieves better performance than YOLOv3. Figure 4 shows the network of ML-YOLOv3.

### CSP-Ghost-ResNet

CSPNet ^30^ is used to solve the problem of complex networks and repeated gradient calculations. The great achievements of computer vision currently rely on expensive hardware equipment, which is difficult to deploy in cheap devices. CSPNet can alleviate the problem of large reasoning calculations and reduce FLOPs and parameter sizes.

CSPNet proves that adding branch paths improves network performance more than broadening or deepening the network. CSPNet ^30^ divides the feature map of the base layer into two parts and merges them through the cross-stage hierarchical structure. The main concept is dividing the gradient flow and making the gradient flow propagate in different network paths. In this way, the gradient information propagated by the network exhibits a large difference in correlation.

CSPNet mainly solves the following problems.

1. The lightweight CNN greatly reduces the accuracy ^30^. CSPNet can maintain sufficient accuracy and ensure light weight. CSPNet can be easily applied to ResNet, ResNeXt ^32^ and DenseNet ^33^. The branch network reduces computational costs. In the field of image classification, CSPNet can achieve the same or higher detection accuracy than the original algorithms.
2. CSPNet can evenly distribute the amount of calculation in each layer of CNN, effectively improve the utilization of each computing unit, and reduce unnecessary redundant parameters. CSPNet reduces PeleeNet's computing bottleneck by half. CSPNet can effectively reduce the computing bottleneck by 80% compared with YOLOv3 ^30^.
3. CSPNet only has few channels for convolution, which effectively reduces memory consumption and reasoning time. For PeleeNet ^34^, the memory consumption after fusion of CSPNet is reduced by 75% ^30^.

**Figure 5:**
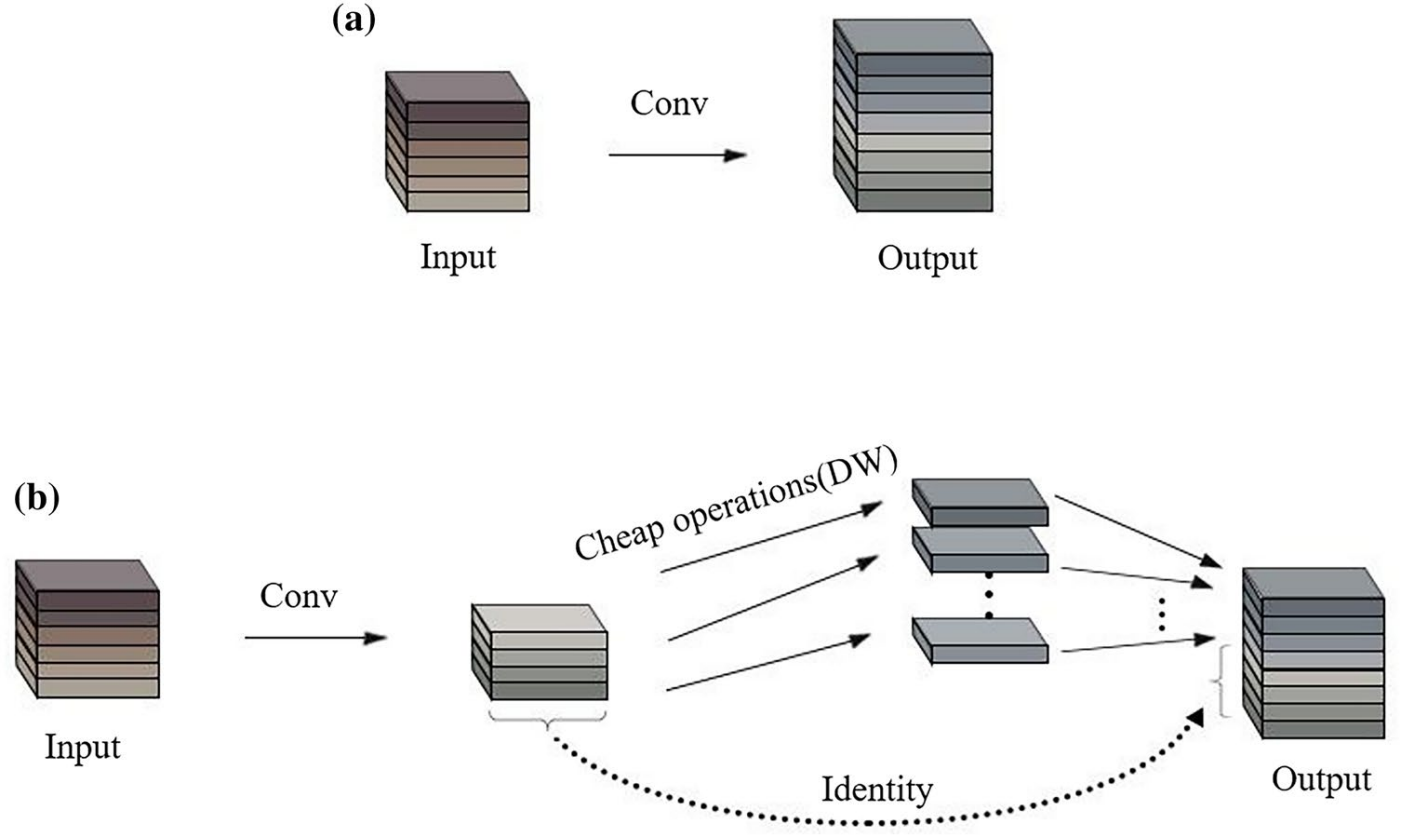
The traditional convolution layer and Ghost module, where (**a**) represents the traditional convolutional layer and (**b**) represents the Ghost module.

GhostNet ^31^ is a new lightweight neural network proposed by HAN et al. GhostNet proposes a Ghost module that replaces the traditional convolutional layer. It generates "ghost" feature maps that can extract the required information from the original features using fewer computational costs. The ghost module divides the traditional convolution into two parts. The first step is performing a 1 $\times$ 1 convolution and outputting a feature map with a smaller number of channels. The second step is generating more feature maps by performing a series of simple linear operations. Figure 5 shows the traditional convolution layer and Ghost module.

Assuming that $X$ represents the input feature map, $H$ and $W$ represent the width and height of the input and output feature maps, respectively, and $n$ represents the number of channels in the input feature map. $Y$ represents the output feature map, and $N$ represents the number of channels of the output feature map. The traditional convolution layer can be represented by Eq. 4.

4 $$Y = X*f + b$$

where “$*$” represents the convolution, $b$ represents the bias, and $f$ represents convolution kernels. Assuming that the size of the convolution kernel is $a \times a$, parameter sizes and FLOPs of the traditional convolution are represented by Eqs. (5),(6).

5 $$P_{1} = a \times a \times n \times N$$

6 $$F_{1} = a \times a \times n \times N \times H \times W$$

The computational costs of traditional convolution are determined by the sizes of the input and output feature maps. There are many similar redundant features generated by traditional convolution, so the traditional convolution layer can waste computing resources. Traditional convolution is replaced by a convolution layer with fewer output feature layers and another linear operation that can cut redundant features and reduce computation cost. Mixing a small number of traditional convolutions together, a lightweight linear operation can reduce the complexity of the network while retaining the key features ^31^.

Assume that the feature map generated by a small amount of traditional convolution is $Z$. $n/2$ represents the number of channels in the output feature map. Traditional convolution in the Ghost module is represented by Eq. 7.

7 $$Z = X*f^{^{\prime}}$$

where $f^{^{\prime}}$ represents convolution kernels. Assuming that the size of the convolution kernel is $a \times a$, the parameter sizes and FLOPs of a small amount of traditional convolution are represented by Eqs. (8)-(9).

8 $$P_{2} = a \times a \times n/2 \times n$$

9 $$F_{2} = a \times a \times n/2 \times n \times H \times W$$

Assume that a simple linear operation is performed on $Z$ to generate a feature map $M$. The linear operation in the Ghost module can be represented by Eq. (10).

10 $$M_{ij} = \Phi_{i,j} \left( {Z_{i} } \right) \forall i,j = 1,2,...,n/2$$

where $Z_{i}$ represents the i-th feature map, $\Phi_{i,j}$ represents the j-th linear operation on the i-th feature map, and $M_{i,j}$ represents the result of the linear operation. Supposing that the size of the convolution kernel is $a \times a$, the parameter sizes and FLOPs of a simple linear transformation operation are represented by Eqs. (11)-(12).

11 $$P_{3} = a \times a \times n/2$$

12 $$F_{3} = a \times a \times n/2 \times H \times W$$

The total parameter sizes and FLOPs generated by the Ghost module are represented in Eqs. (13)-(14).

13 $$P_{4} = a \times a \times n/2 \times n + a \times a \times n/2$$

14 $$F_{4} = a \times a \times n/2 \times n \times H \times W + a \times a \times n/2 \times H \times W$$

Under the premise of the same feature size, the parameter sizes and FLOPs required in the Ghost module have been reduced compared with the traditional convolutional network. Compared with other lightweight methods, the Ghost module achieves a good detection effect and effectively reduces the calculation cost. Based on the Ghost module, HAN et al. established an efficient neural network GhostNet ^45^. Experiments show that GhostNet is superior to advanced efficient depth models such as MobileNet and makes rapid inferences on mobile devices.

**Figure 6:**
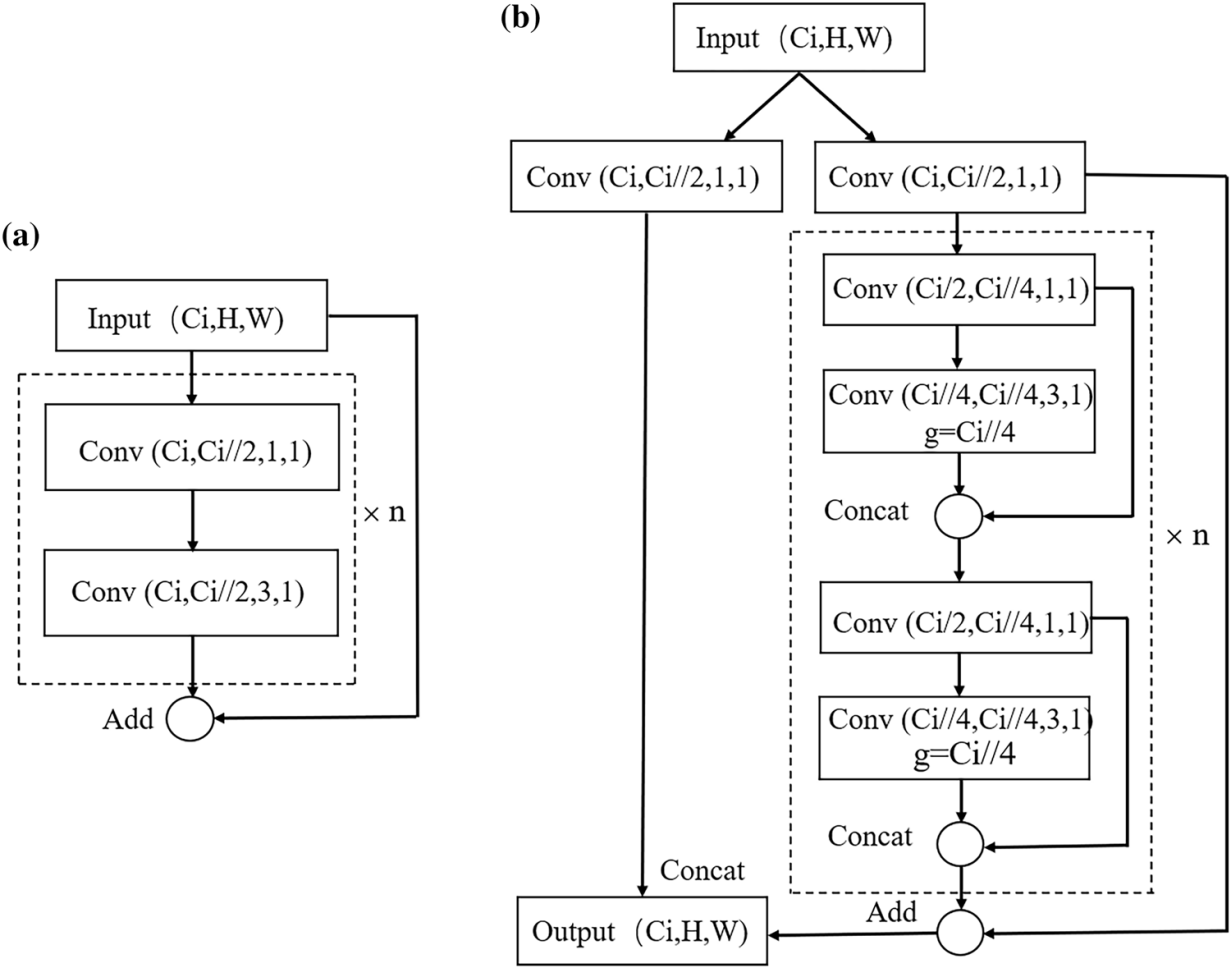
Network of ResNet and CSP-Ghost-ResNet. (**a**) represents the network of ResNet, and (**b**) represents the network of CSP-Ghost-ResNet.

Both CSPNet and GhostNet show excellent feature extraction capabilities. Therefore, the paper integrates the two networks and proposes a new residual network named CSP-Ghost-ResNet. Different from ResNet, the overall architecture of CSP-Ghost-ResNet refers to CSPNet, and the stacked network is changed to the Ghost module. Figure 6 shows the ResNet and CSP-Ghost-ResNet networks.

YOLOv3 uses the ResNet network and stacks the network in the dashed box. We name the network inside the dashed box the stacking module. The ResNet backbone network first performs 1 $\times$ 1 traditional convolutions on the input feature map and then performs 3 $\times$ 3 traditional convolutions. ML-YOLOv3 uses the CSP-Ghost-ResNet network and replaces the stacked traditional convolution with double Ghost modules. CSP-Ghost-ResNet first shunts input features. It uses 1 $\times$ 1 traditional convolution to reduce the number of channels by half and divides the input features into two channels (Part 1 and Part 2). Part 1 does not perform convolution. The convolution of the Ghost module is stacked twice in Part 2. The feature map that is generated by Ghost modules and the input feature of Part 2 are connected to form the output feature of Part 2 through “add”. Finally, the output features of Part 1 and Part 2 are connected through “concat”.

FLOPs and parameter sizes of the first stacking module for YOLOv3 and ML-YOLOv3 are calculated. The size of the input feature map of YOLOv3 is 208 $\times$ 208 $\times$ 64. ML-YOLOv3 reduces the number of channels by half through the CSPNet network. Therefore, the input feature map size of the stacking block in ML-YOLOv3 is 208 $\times$ 208 $\times$ 32. Tables 1 and 2 show the results of YOLOv3 and ML-YOLOv3, respectively.

**Table 1:** FLOPs of the first stacking module of the Residual block and CSP-Ghost-ResNet.

	Residual block (MB)	CSP-Ghost-ResNet (MB)
1 × 1 convolution	338	169
3 × 3 convolution	3042	47.53
Total	3380	216.53

**Table 2:** Parameter size of the first stacking module of the Residual block and CSP-Ghost-ResNet.

	Residual block (KB)	CSP-Ghost-ResNet (KB)
1 × 1 convolution	8	4
3 × 3 convolution	72	1.125
Total	80	5.125

From Tables 1 and 2, the stacking module used in ML-YOLOv3 reduces parameter sizes and FLOPs by 93.59% compared with YOLOv3. This is mainly because only 1/2 of the channels are involved in the convolution. In addition, the traditional 3 $\times$ 3 convolution is abandoned, and depthwise separable convolution is used to extract image features, which greatly reduces the computational cost and ensures the detection effect.

### ML-Darknet

**Figure 7:**
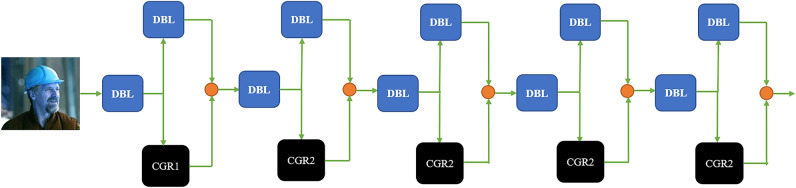
Network of ML-Darknet.

Darknet53 is a network with a complex structure and a large number of parameters. It adopts the method of direct connection of downsampling and the residual network. Although this connection method is beneficial to extract features, it also generates high computational cost. CSPNet has demonstrated excellent performance in many networks. We also described the many benefits of CSPNet above. Based on this, we design a lightweight network, ML-Darknet, by fusing CPSNet and Darknet53. Figure 7 shows the network results of ML-Darknet.

In Fig. 7, ML-Darknet adopts the network structure of downsampling to connect CSPNet modules so that only half of the channels participate in the CSP-Ghost-ResNet operation. In addition, CSP-Ghost-ResNet also adopts CSPNet modules, and the channels actually participating in the operation of the stacking module are only 1/4 of the input channels. In ML-Darknet, CSP-Ghost-ResNet’s number of each residual network is 1, 2, 2, 2, 2. This simplifies the number of stacking modules. Such a network can effectively reduce the complexity of the model.

We compute FLOPs and parameter sizes for the first stacking module of ML-Darknet. The operation results are shown in Table 3. Compared with Darknet53, the FLOPs and parameter sizes of the first stacking module of ML-Darknet are reduced by 96.80%. Compared with the downsampling connection CSP-Ghost-ResNet, the FLOPs and parameter sizes of the first stacking module of ML-Darknet are reduced by 50%. The branched network adopted by ML-Darknet widens the backbone network, resulting in a small computational cost. However, it drastically reduces the computational cost of stacking modules. Experiments show that ML-Darknet effectively reduces the computational cost of the backbone network.

**Table 3:** FLOPs and parameter sizes of the first stacking module of ML-YOLOv3.

	FLOPs (MB)	Parameter sizes (KB)
1 × 1 convolution	84.5	2
3 × 3 convolution	23.765	0.5625
Total	108.265	2.5625

### PAN-CGR-Network

**Figure 8:**
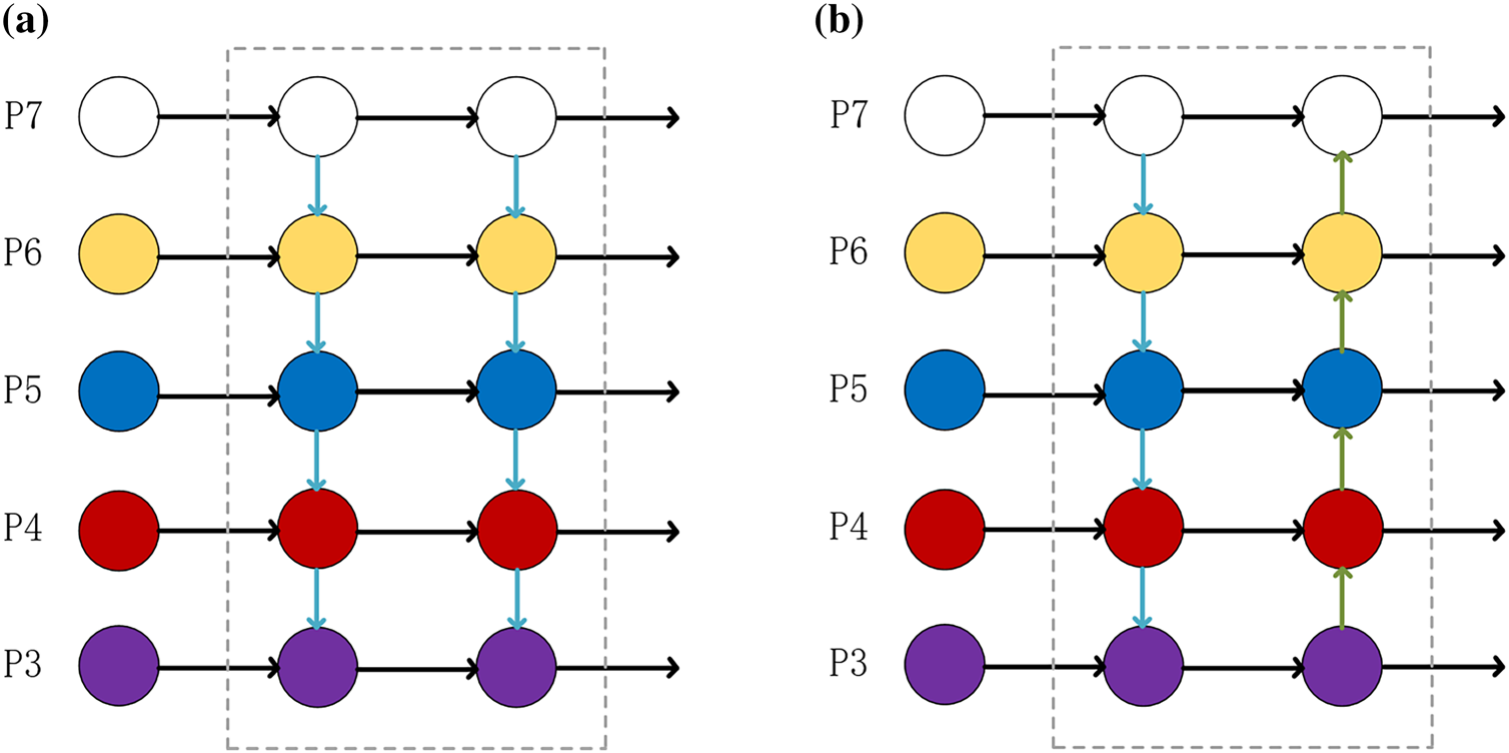
The backbone network of FPN and PANet.

The path of the multiscale feature extraction network of Mask R-CNN is too long, which increases the difficulty of accurately locating information. Based on this problem, Liu Shu et al.^22^ proposed PANet. PANet is one of the mainstream object detection and segmentation networks. It improves the traditional backbone network structure, proposes bottom-up feature fusion, and reinforces the feature pyramid twice; at the same time, it performs a pixel-by-pixel sum operation during feature fusion, shortening the fusion distance of high- and low-level features. In addition, PANet adds a fully connected branch to the mask branch, which effectively improves the feature extraction ability. PANet proves its superiority in target detection algorithms, such as YOLOv4 and YOLOv5. YOLOv3's multiscale feature extraction network uses FPN, FPN only uses top-down feature fusion methods, and the overall performance is not as good as that of PANet. Figure 8 shows the backbone network of FPN and PANet. In Fig. 8, (a) represents the FPN network and (b) represents the PANet network.

Shallow feature maps contain more detailed features, and deep feature maps contain more semantic features. YOLOv3 can retain most of the image features. However, as the network deepens, some detailed features are still lost in the image, which makes the object positioning insufficiently accurate. Based on this, this paper integrates PANet and CSP-Ghost-ResNet to design a lightweight PAN-CGR-Network.

**Figure 9:**
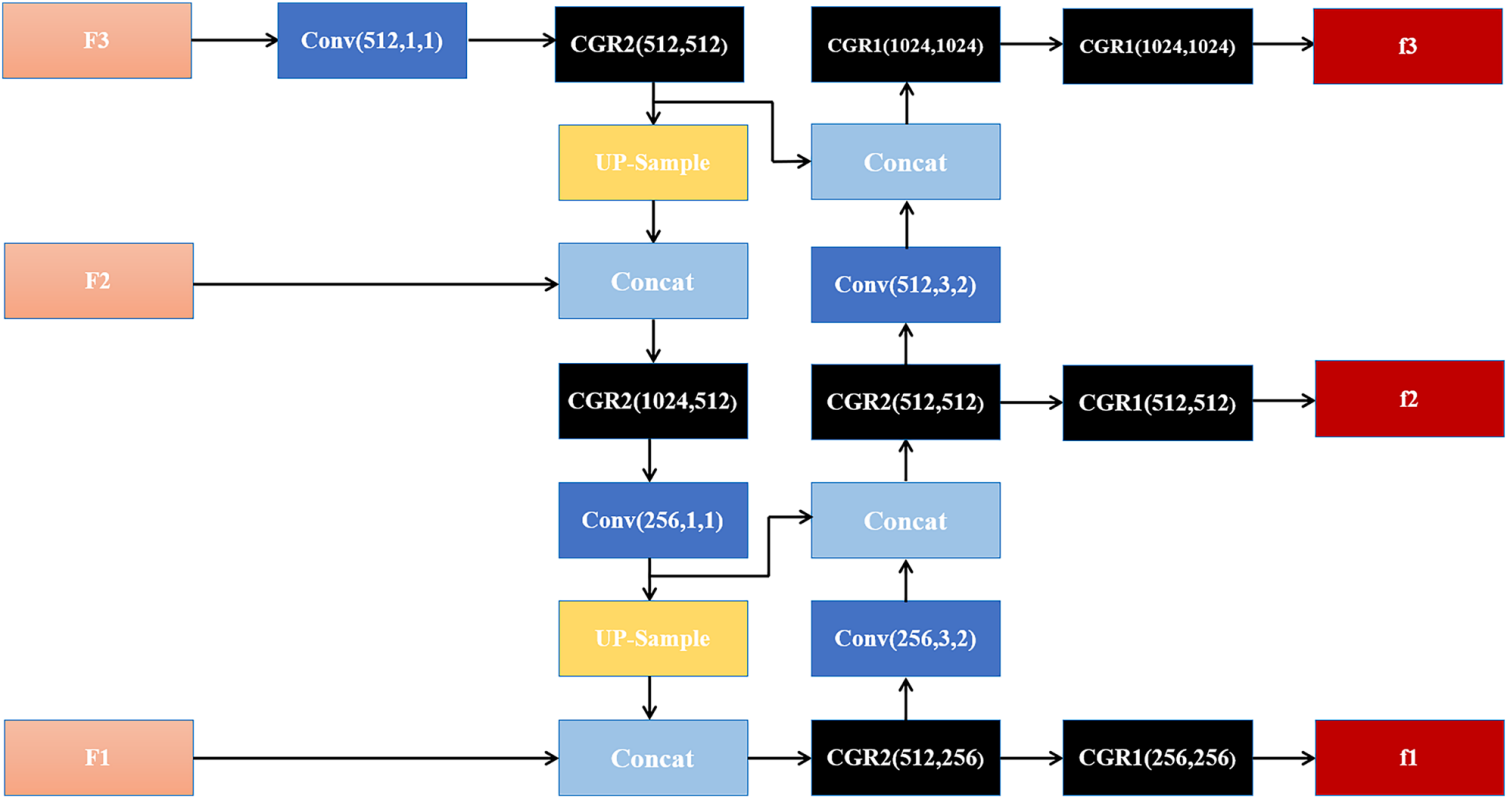
The network of PAN-CGR-Network.

Compared to the FPN, the PAN-CGR-Network adds from bottom-up feature fusion, enhances the fusion of deep features and shallow features, and improves the entire feature hierarchy. In addition, Pan-CGR-Network did not stack a large amount of traditional convolutional structures but used the CSP-Ghost-ResNet lightweight network to significantly reduce the calculation cost of the network. The network of PAN-CGR-Network is shown in Fig. 9.

## Experimental analysis

### Dataset

With the rapid economic development of the world, an increasing number of modern metropolises have emerged. The construction of infrastructure, such as buildings, bridges, and railways, requires a large number of infrastructure workers. In recent years, the number of casualties caused by dangerous operations has increased, although wearing safety helmets can effectively reduce the occurrence of safety accidents. Conventional safety helmet detection mainly occurs through human supervision or video surveillance. Manual detection has lower efficiency, higher cost, and results in missed detection. Video surveillance relies on people looking for abnormal information in massive surveillance videos, and it is difficult to uninterruptedly discover illegal operations over the long term and forbid them in a timely manner. The paper selects safety helmets as the detection object and uses deep learning algorithms to replace manual detection to realize intelligent supervision. In the image and video sequences, “hat” is displayed if the helmet is detected, and “danger” is displayed if the helmet is not detected.

**Figure 10:**
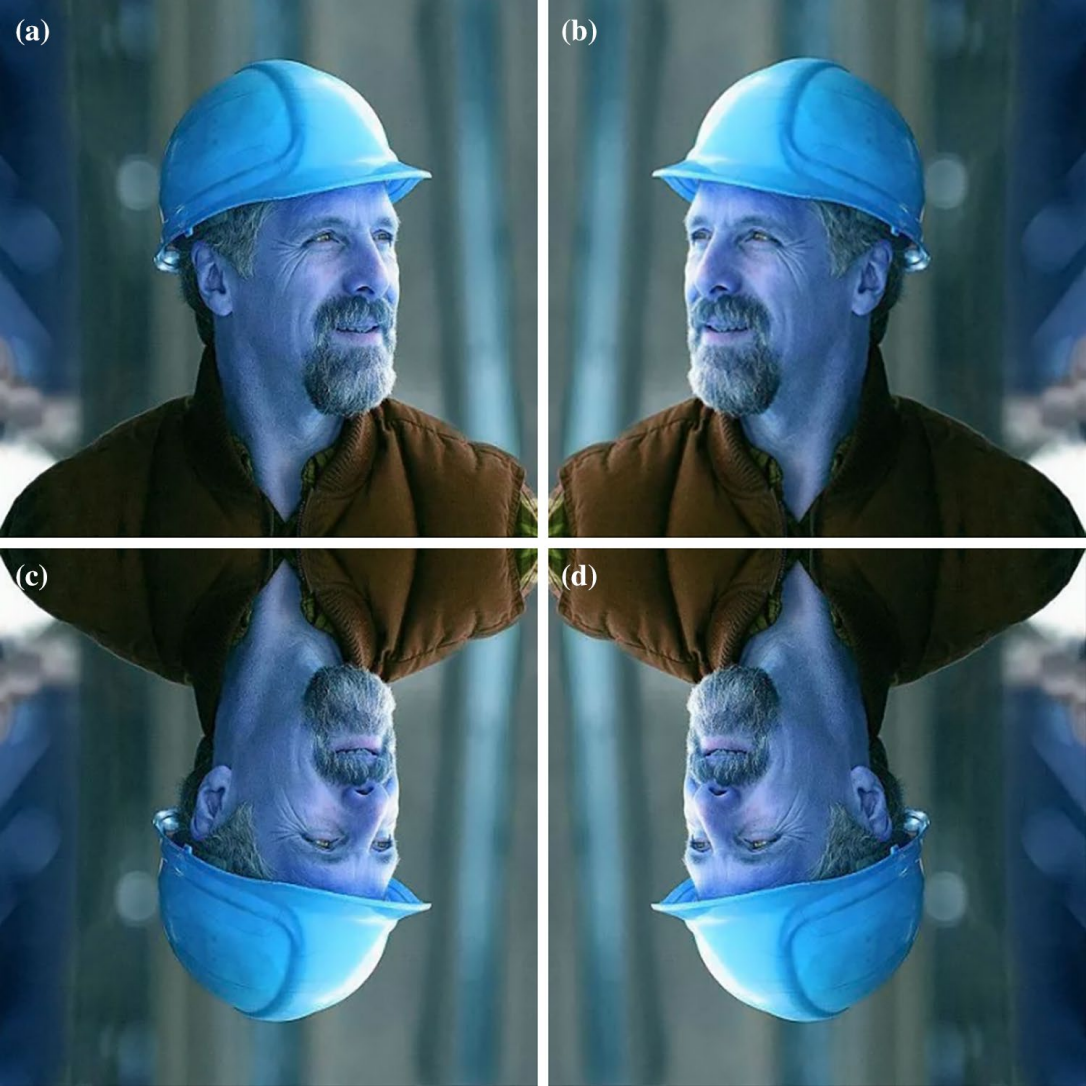
Preprocessing results of the image: (**a**) represents the initial image, (**b**) represents the image after horizontal rotation, (**c**) represents the image after vertical rotation, and (**d**) represents the image after horizontal and vertical rotation.

In the research of deep learning, the quality of the dataset will directly affect the quality of the network. The safety helmet datasets in the paper contain 7,581 images from different application scenes. Images are in an online open-access publication (https://github.com/njvisionpower/Safety-Helmet-Wearing-Dataset). And all images are not modified. The paper preprocesses the existing datasets to improve the training effect of the network. We use data enhancement techniques to improve the generalization of the model. Data enhancement can allow limited data to generate more data value without increasing the size of the data. Data enhancement can be divided into supervised data enhancement and unsupervised data enhancement. Among them, supervised data enhancement includes enhancement of single sample data and diverse sample data enhancement; unsupervised data enhancement includes new data and learning enhancement strategies. Instead of using overly complex enhancement methods, we only use geometric transformation to expand the dataset for a single sample, including horizontal rotation, vertical rotation, horizontal rotation and vertical rotation. Figure 10 shows the preprocessing results for the dataset.

Meanwhile, the input size of the dataset is normalized, and the input feature map is adjusted to 416 $\times$ 416. Both ML-YOLOv3 and YOLOv3 use three feature maps of different scales for target detection. The paper uses the K-means method to generate 9 anchors with different sizes, and the feature map of each scale distributes 3 anchors. NMS is added to the end of the network to retain more accurate prediction results.

### Detection results of ML-YOLOv3

**Figure 11:**
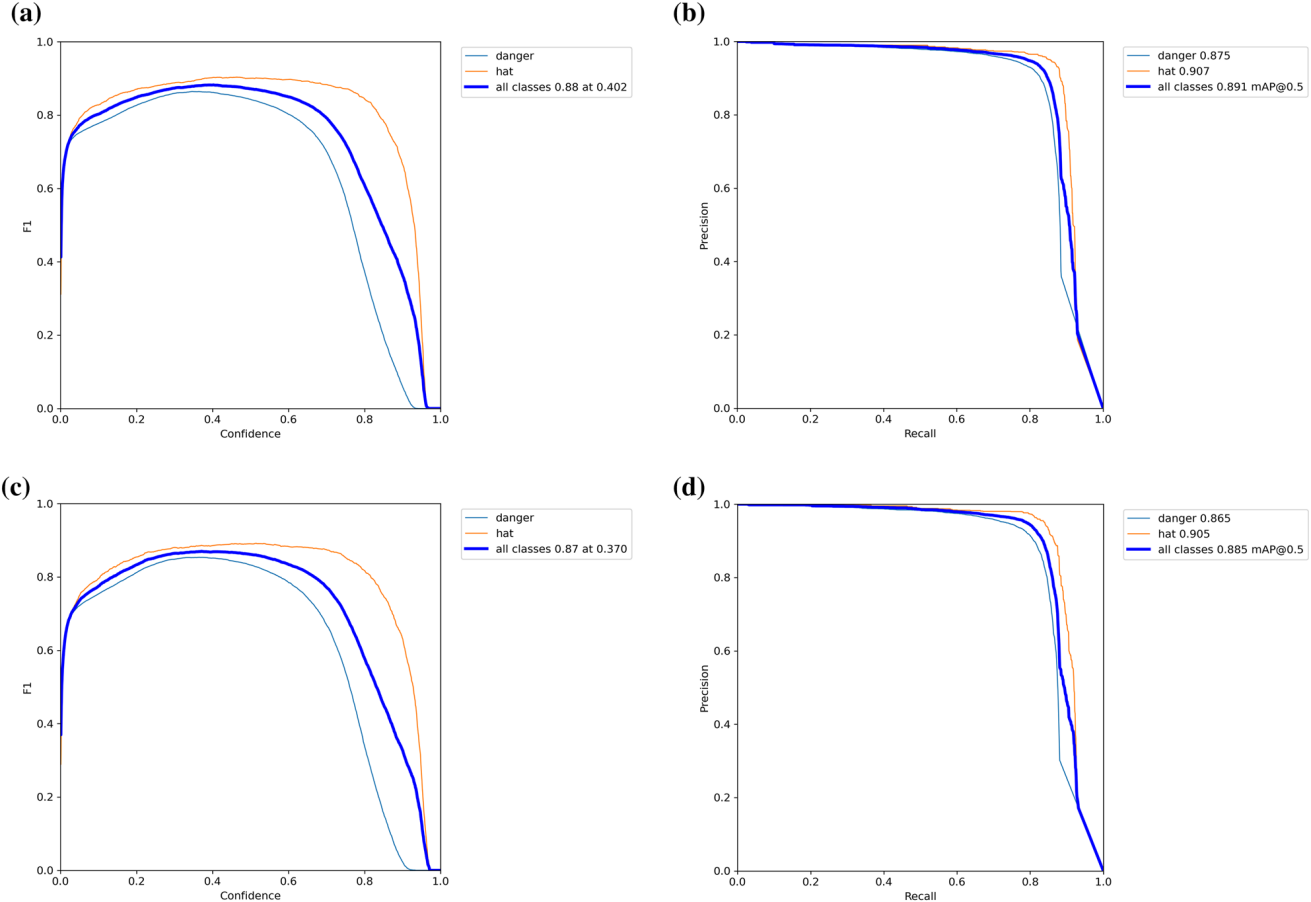
F1-scores and P-R curves for the two methods.

**Figure 12:**
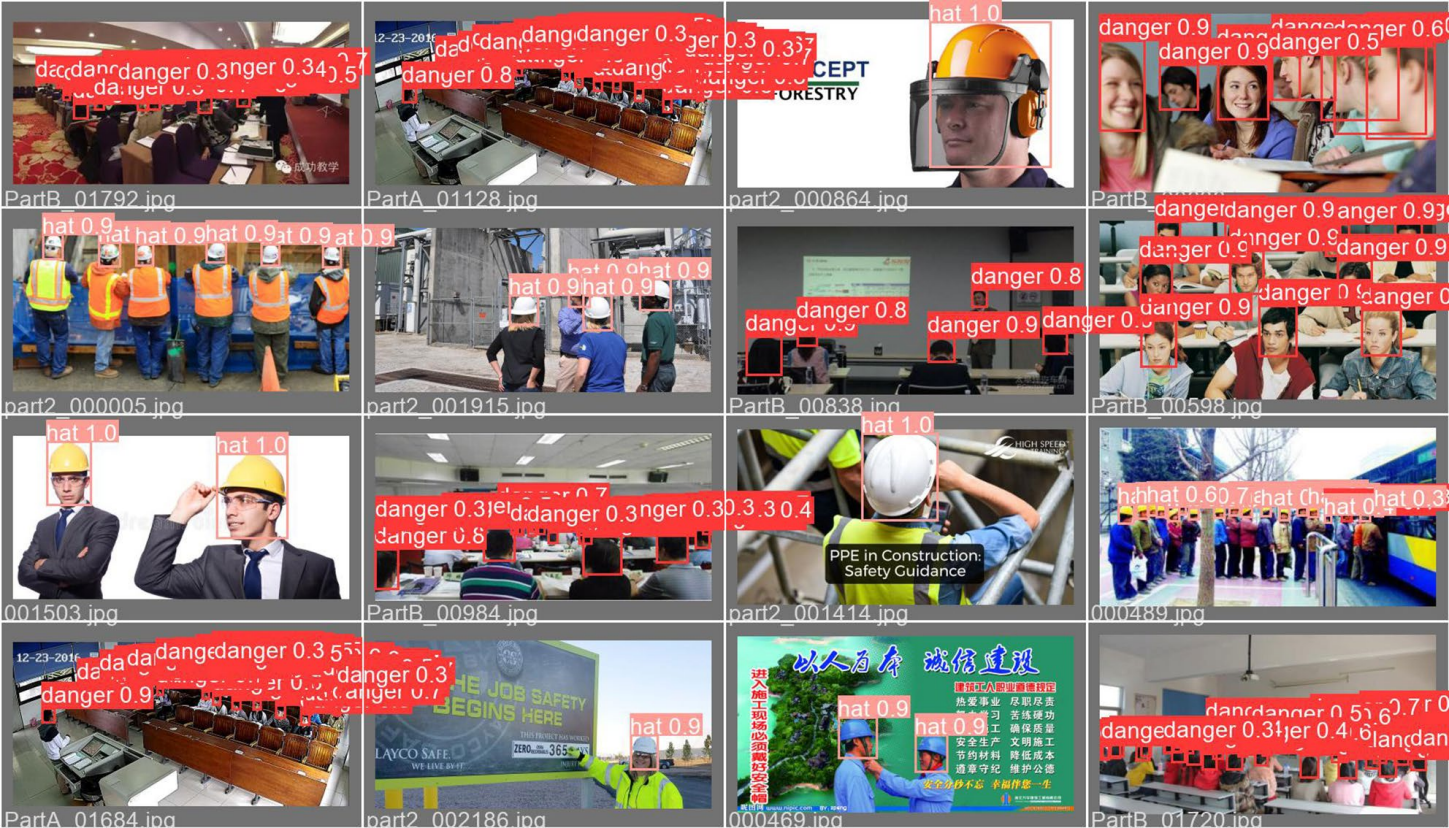
The detection results of ML-YOLOv3s.

ML-YOLOv3 is a lightweight version of YOLOv3. The detection effects of ML-YOLOv3 and YOLOv3 are compared using the safety helmet dataset. Figure 11 shows the P-R curves and F1 scores of the two methods, where (a) and (b) are the F1 score and P-R curve of YOLOv3, respectively, and (c) and (d) are the F1 score and P-R curve of ML-YOLOv3, respectively. It is undeniable that YOLOv3 performs better than ML-YOLOv3, but the gap between the two is not large. In practical engineering applications, hardware devices will have less impact on the performance of ML-YOLOv3 because ML-YOLOv3 has a smaller computing cost and model volume. Figure 12 shows the detection results of ML-YOLOv3. In addition, our improved methods yield good detection results. The detection effect of these improved methods can be found in Supplementary Figs. S1−S6 online.

### Ablation Experiments

We redesigned the YOLOv3 network and proposed three network improvements. To prove the impact of the three improved methods on the performance of the model and avoid inappropriate improvement and reduced network performance, we conducted ablation experiments based on the helmet dataset. First, in the detection effort, the paper compares the influences of different improved methods on the mAP. Second, the paper makes a comparison of other indicators that influence the complexity of the model. YOLOv5 is the mainstream object detection algorithm. Therefore, in the experimental analysis stage, this paper not only compares our improved method with YOLOv3 and YOLOv3-Tiny: we also added its comparison with YOLOv5s, YOLOv5n, YOLOv5l and YOLOv5l in terms of computing cost and detection effect, reflecting the contribution of our proposed lightweight algorithm.

**Figure 13:**
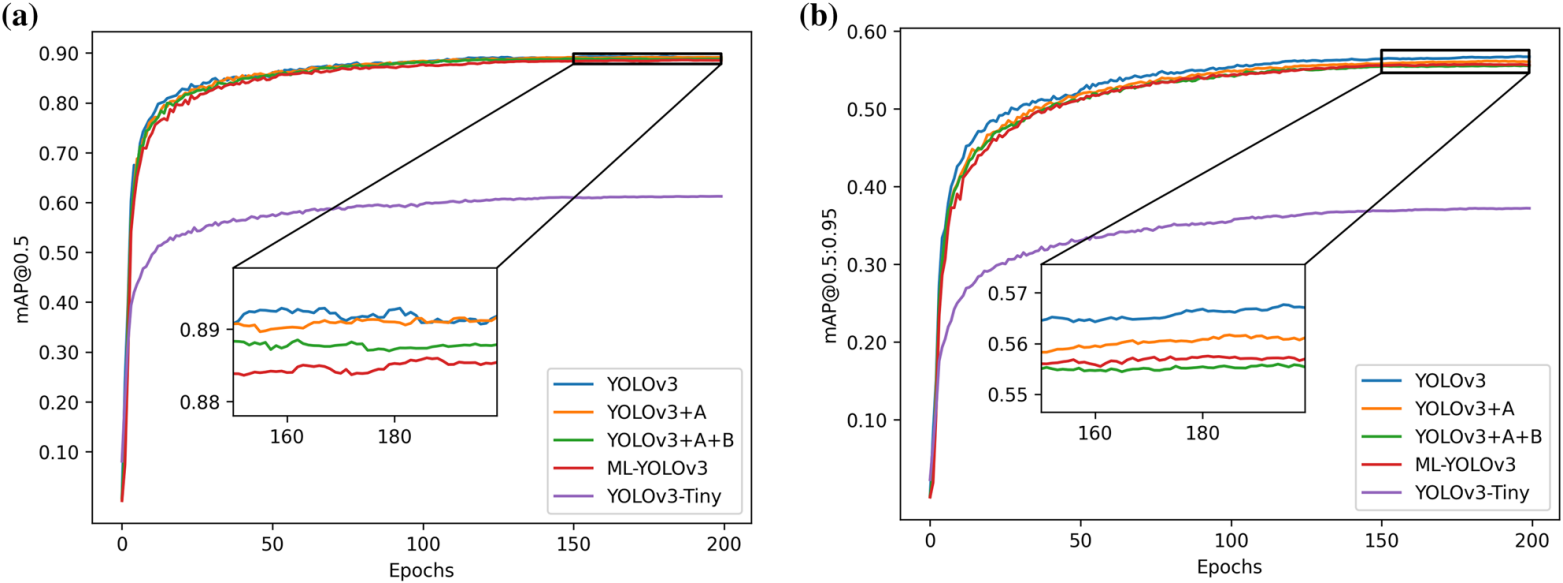
The comparison results between our improvement and YOLOv3 and YOLOv3-tiny in terms of the detection effect.

**Figure 14:**
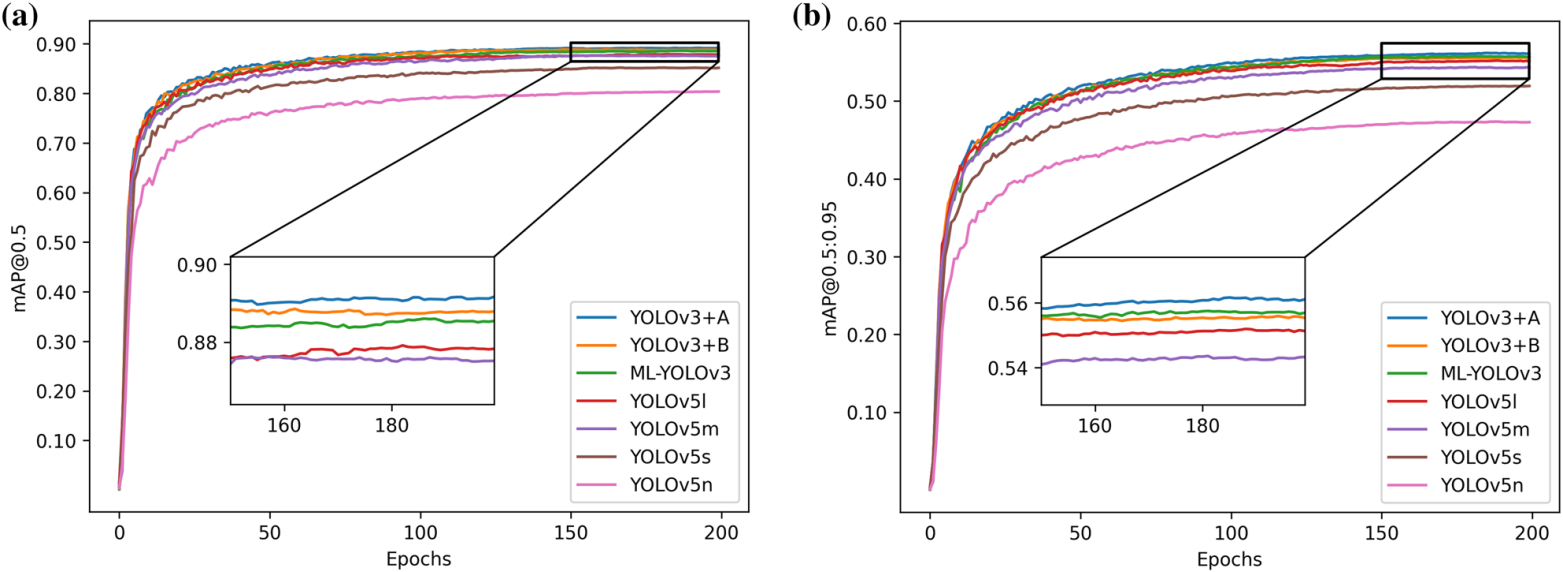
The comparison results of our improvement and YOLOv5s, YOLOv5n, YOLOv5l and YOLOv5l in terms of the detection effect.

In terms of the detection effect, mAP@0.5 and mAP@0.5:0.95 are used as the evaluation indices to compare our improved method with other algorithms. The number after ‘‘@” represents the specific threshold of IOU. Figure 13 shows the comparison results between our improvement and YOLOv3 and YOLOv3-tiny in terms of the detection effect. Figure 14 shows the comparison results of our improvement and YOLOv5s, YOLOv5n, YOLOv5l and YOLOv5l in terms of the detection effect, where “A” represents CSP-Ghost-ResNet and “B” represents ML-Darknet. In Fig. 13, “YOLOv3 + A”, “YOLOv3 + A + B” and ML-YOLOv3 perform worse than YOLOv3 with respect to the two indicators. The result is also within our expected range. The lightweight model reduces the parameter sizes for training. An excellent network improves the utilization of parameters and makes the model more lightweight, but it is not better than YOLOv3 in the optimization of the model. This is because YOLOv3 has a large parameter size. However, what is undeniable is that we improve the method of detecting the effect, and YOLOv3 does not exhibit a very large difference. Comparing YOLOv3-Tiny, the improved method is significantly better than YOLOv3-Tiny. In Fig. 14, the performances of “YOLOv3 + A”, “YOLOv3 + A + B” and ML-YOLOv3 under the two indicators are superior to that of YOLOv5, which fully demonstrates the effectiveness of the proposed improvement method.

ML-YOLOv3 aims to significantly reduce the calculation cost of the model on the premise of maintaining excellent detection effort. Academic studies are more accustomed to attaching importance to the detection effect of the model and ignoring other aspects of the performance. However, in practical applications, the delay caused by hardware equipment will affect the detection effect, and a lightweight network model can more effectively overcome the impact of hardware equipment. Therefore, we do not pay much attention to the subtle changes in the detection effect, but instead devote more attention to the performance effect of the improved method in the calculation cost. We performed ablation experiments on the FLOPs, parameter sizes, speed (model inference time) and frames per second (FPS). Table 4 shows the comparison results of calculation cost between our improved method and other algorithms. In addition, the detection effects of various algorithms are summarized, and the best effects of various algorithms are recorded in Table 5.

**Table 4:** Results of ablation experiments on some lightweight indicators.

Algorithm	FLOPs (GB)	Parameters (MB)	Speed (ms)	FPS
YOLOv3-Tiny	12.9	8.7	3.2	238
YOLOv3	154.9	61.5	17.2	55
YOLOv5n	4.2	1.8	3.8	208
YOLOv5s	15.9	7.0	5.6	152
YOLOv5m	48.0	20.9	8.2	109
YOLOv5l	107.8	46.1	13.2	70
YOLOv3 + A	68.9	30.8	10.6	86
YOLOv3 + A + B	65.2	29.5	10.1	90
ML-YOLOv3	46.0	18.1	9.7	93

**Table 5:** Results of ablation experiments on AP and mAP.

Algorithm	AP@0.5 (%) danger	AP@0.5 (%) hat	mAP@0.5 (%)	AP@0.5:0.95 (%) danger	AP@0.5:0.95 (%) hat	mAP@0.5:0.95 (%)
YOLOv3-Tiny	44.8	77.6	61.2	20.9	53.4	37.2
YOLOv3	87.5	90.7	89.1	44.8	68.7	56.8
YOLOv5n	75.8	85.0	80.4	34.7	59.8	47.3
YOLOv5s	81.6	88.7	85.1	39.5	64.4	52.0
YOLOv5m	84.9	90.0	87.5	42.2	66.5	54.4
YOLOv5l	85.8	89.8	87.8	43.1	67.2	55.2
YOLOv3 + A	87.1	91.1	89.1	44.2	68.2	56.2
YOLOv3 + A + B	86.7	90.9	88.8	43.8	67.5	55.7
ML-YOLOv3	86.5	90.5	88.5	43.6	67.9	55.8

“@’’ indicates the IOU threshold parameter.

As shown in Tables 4 and 5, the improved method proposed in this paper has obvious advantages. The detection effect of “YOLOv3 + A” is better than that of YOLOv5. In mAP@0.5, mAP@0.5:0.95 and FPS, “YOLOv3 + A” was 1.3%, 1.0% and 16 frames better than YOLOv5l, respectively. FLOPs, parameter size and speed of “YOLOv3 + A” decreased by 38.9 GFLOPs, 15.3 MB and 2.6 ms, respectively. In addition, mAP@0.5 of “YOLOv3 + A” achieves the same effect as YOLOv3, but with FLOPs, parameter size and speed reduced by 86.0 GFLOPs, 30.7 MB and 6.6 ms, respectively. The computational cost of “YOLOv3 + A + B” is further reduced. Compared with “YOLOv3 + A”, its FLOPs, parameter size and speed are reduced by 3.7 GFLOPs, 1.3 MB and 0.5 ms, respectively. While the model is lightweight, the detection effect decreases slightly. However, the detection effect of “YOLOv3 + A + B” is still superior to that of YOLOv5. On the basis of “YOLOv3 + A + B”, ML-YOLOv3 redesigns the multiscale feature extraction network, which greatly reduces the computational cost of the model. The FLOPs, parameter sizes and speed of ML-YOLOv3 are only 29.7%, 29.4% and 56.4% of those of YOLOv3, but mAP@0.5 and mAP@0.5:0.95 are only 0.6% and 1.0% lower. Compared with YOLO5, the calculation cost of ML-YOLOv3 is lower than that of YOLOv5m, but the detection effect surpasses that of YOLOv5l. The lightweight versions in the table, such as YOLOv3-Tiny, YOLOv5n and YOLOv5s, greatly reduce the detection effect while reducing the model cost. The improved method proposed in this paper not only ensures the detection effect but also effectively reduces the calculation cost of the model.

## Conclusions

The large network of YOLOv3 affects applications in mobile or cheap devices. The paper proposes a lightweight object detection network: ML-YOLOv3. In this paper, three network improvement methods are proposed, which can significantly reduce the computational cost of the model while maintaining a strong detection effect. Based on the helmet dataset, CSP-Ghost-ResNet proposed by us effectively reduces the complexity of the model and achieves almost the same level of detection effect as YOLOv3. ML-Darknet reduces the detection effect of the model, but it effectively reduces the computational cost of the model. In addition, PAN-CGR-Network is redesigned in this paper. It further reduces computing costs. Experiments have shown that the FLOPs, parameter sizes and speed of ML-YOLOv3 are only 29.7%, 29.4 and 56.4% of those of YOLOv3. Compared with YOLOv5, ML-YOLOv3 achieves better detection performance than YOLOv5l with lower computational cost than YOLOv5m. Ml-YOLOv3 balances the detection effect and calculation cost and surpasses the mainstream object detection algorithm in terms of some indicators.

## Future prospects

Thus far, the work performed in this paper has been introduced. In this paper, a lightweight target detection algorithm, ML-YOLOv3, is designed. ML-YOLOv3 takes into account the detection effect and calculation cost and offers some advantages over the current efficient object detection algorithm. In addition, we provide a model optimization idea for relevant scholars. We believe that in some scenarios, reducing the calculation cost of the model is more practical than improving the accuracy. Especially with the popularity of mobile devices, the lightweight model is more suitable for running on such devices.

However, there are still some deficiencies in the improvement of this paper. We reduced the calculation costs of the model and lost a small amount of accuracy. Compared with the optional features of multiple versions of YOLOv5, ML-YOLO3 lacks flexibility. We will continue to work in the field of deep learning. At the same time, we hope that relevant scholars will pay more attention to the lightweight nature of the model and the detection of small targets. We will also focus on these two directions.

## Supplementary Information

Supplementary Information.

## Data Availability

All data generated or analyzed during this study are included in this published article (and its Supplementary Information files).
