# Supplementary material for: A lightweight YOLOv3 algorithm used for safety helmet detection
Source: Sci Rep. 2022 Jun 29;12:10981. doi: 10.1038/s41598-022-15272-w (PMC9243020; doi:10.1038/s41598-022-15272-w)
Supplement: Supplementary file 1 — Supplementary Information. [file 41598_2022_15272_MOESM1_ESM.pdf]

A lightweight YOLOv3 algorithm used for safety helmet detection

Lixia Deng[1*], Hongquan Li[1], Haiying Liu[1], Jason Gu[2]
[1] **S**chool of Information and Automation Engineering, Qilu University of Technology(Shandong Academy of Sciences), Jinan, Shandong Province 250353, China
[2] Department of Electrical and Computer Engineering, Dalhousie University, Halifax, Nova Scotia, Canada

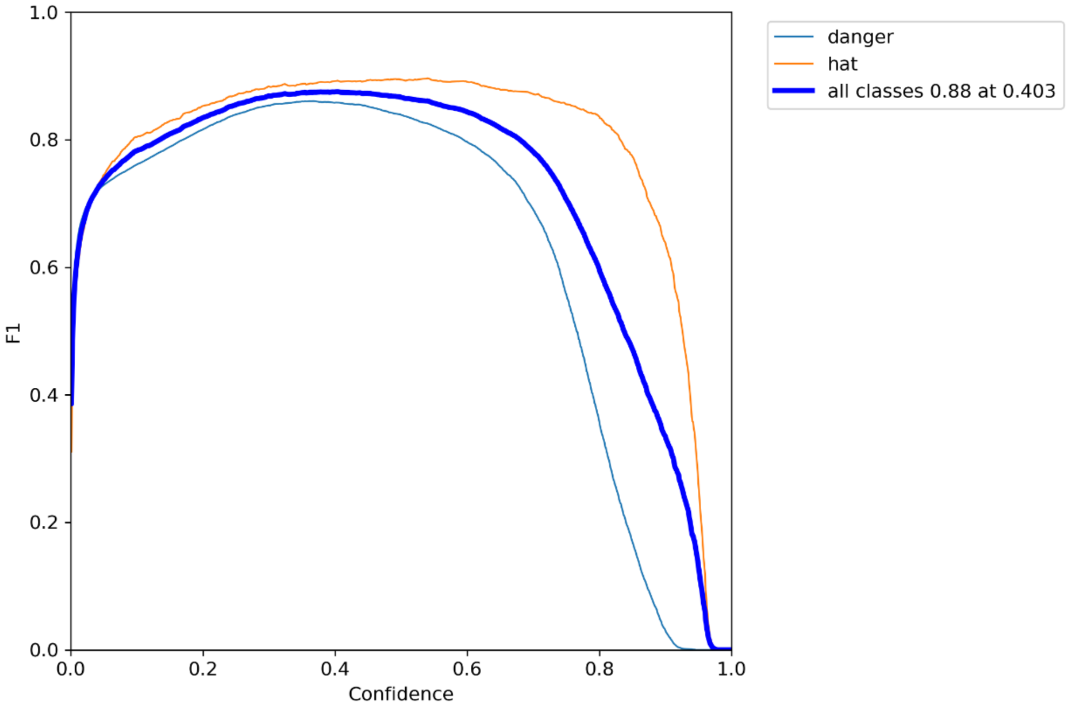

Figure S1. F1 score result of “YOLOv3+A”.

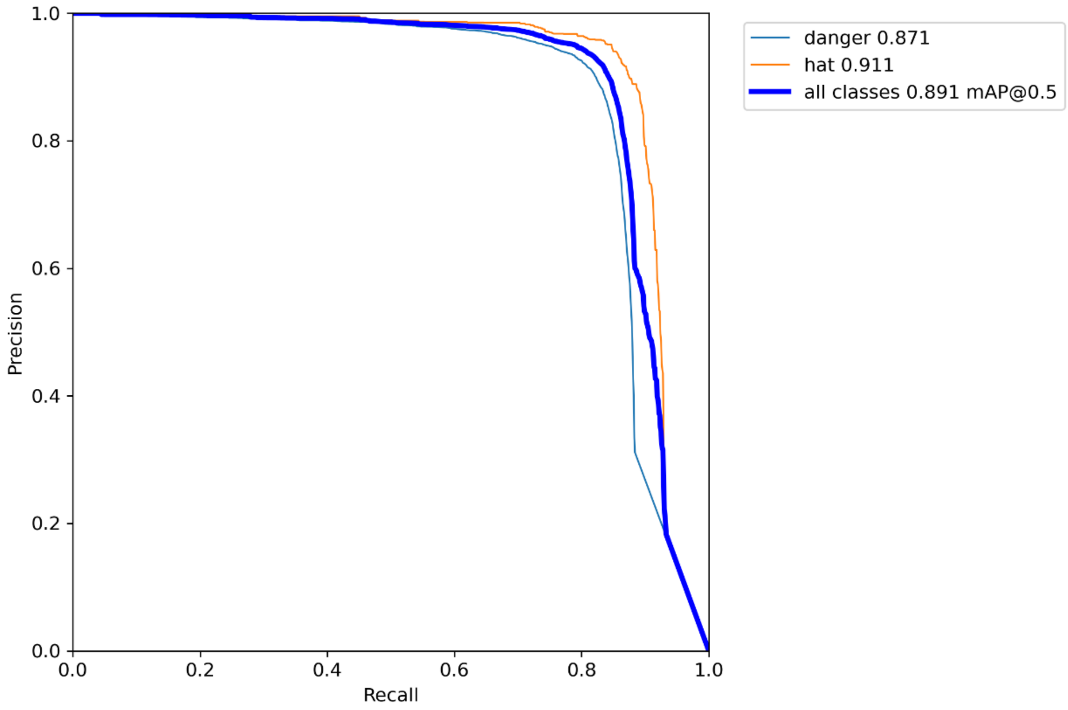

Figure S2. P-R curve results of "YOLOv3+A".

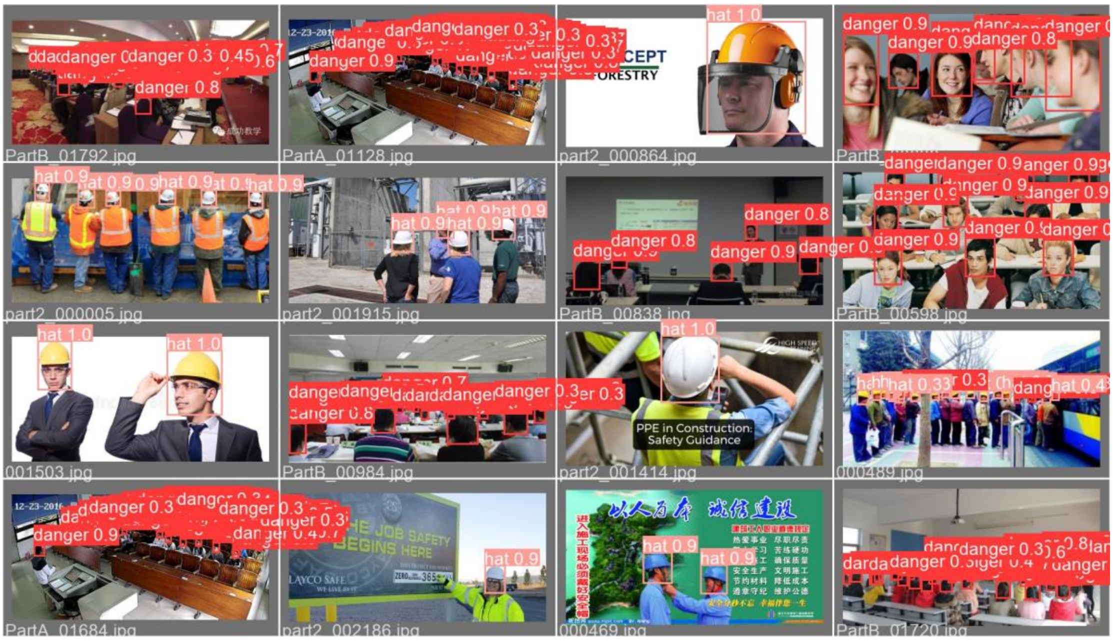

Figure S3. The detection result of "YOLOv3+A".

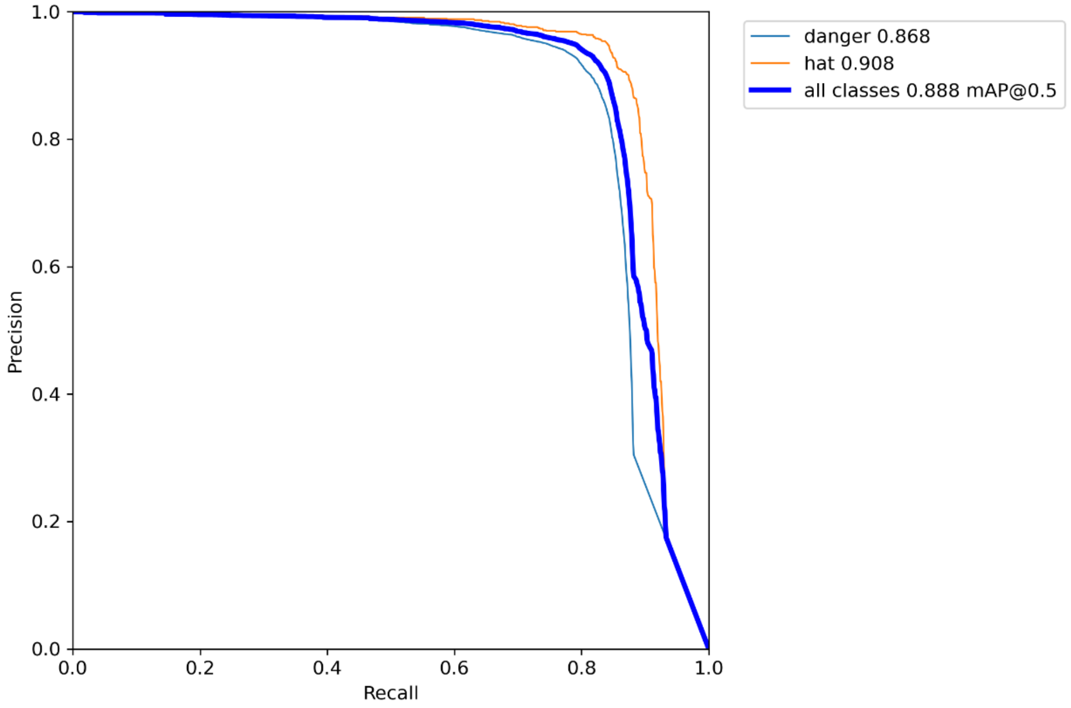

Figure S4. F1 score result of “YOLOv3+A+B”.

Figure S2.

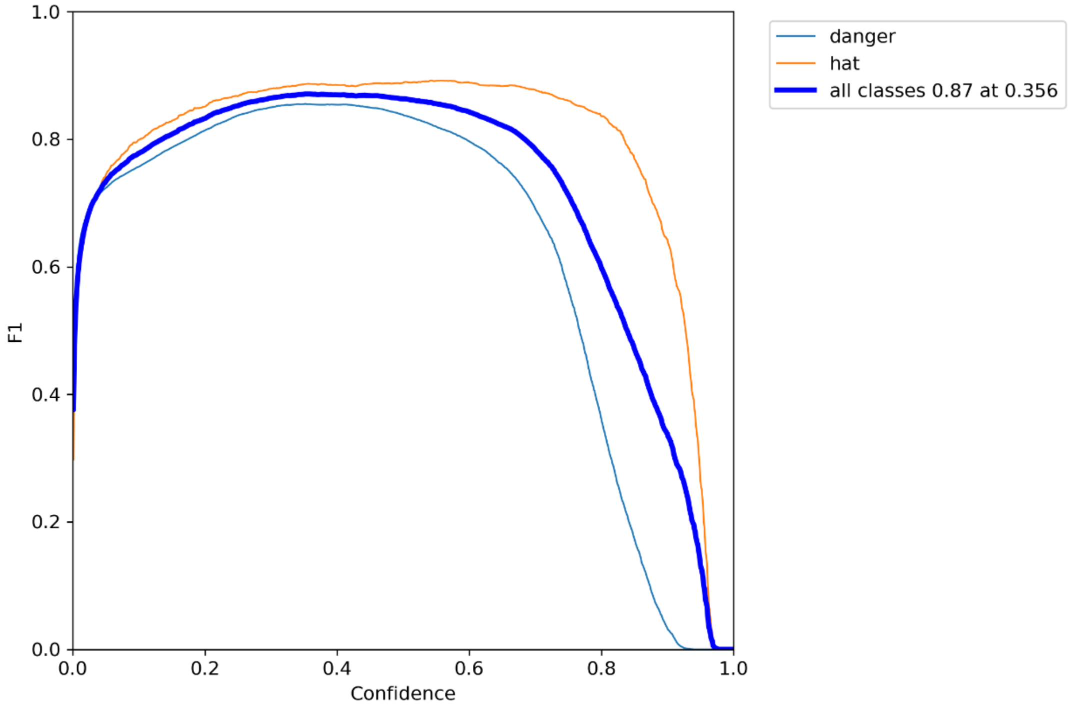

Figure S5. P-R curve results of “YOLOv3+A+B”.

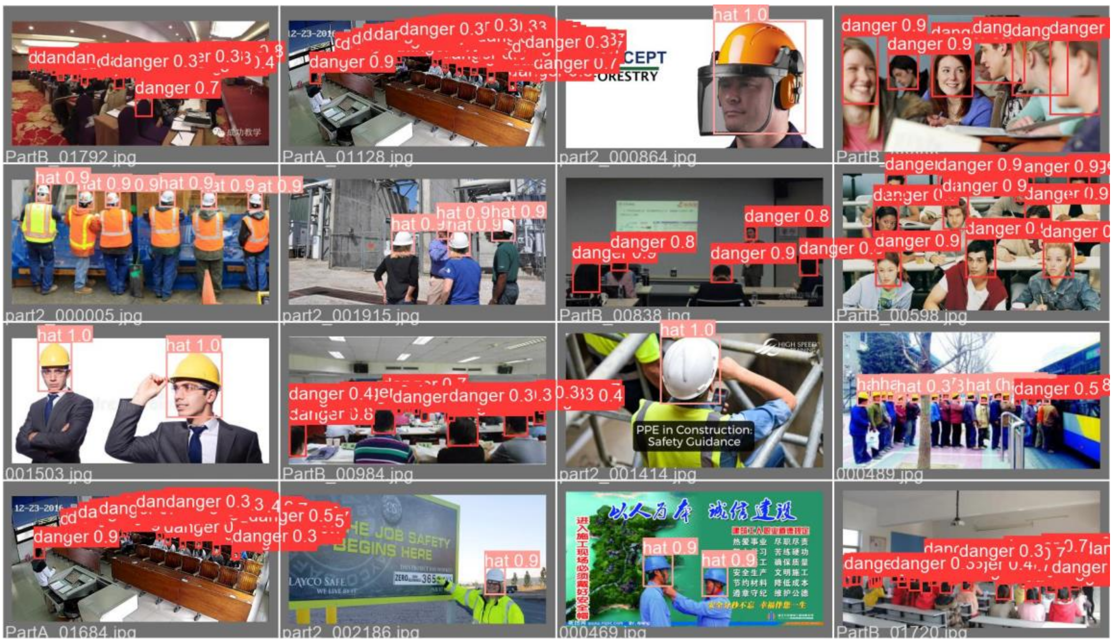

Figure S6. The detection result of “YOLOv3+A+B”.
